# CD73 blockade enhances the local and abscopal effects of radiotherapy in a murine rectal cancer model

**DOI:** 10.1186/s12885-020-06893-3

**Published:** 2020-05-12

**Authors:** Hidenori Tsukui, Hisanaga Horie, Koji Koinuma, Hideyuki Ohzawa, Yasunaru Sakuma, Yoshinori Hosoya, Hironori Yamaguchi, Kotaro Yoshimura, Alan Kawarai Lefor, Naohiro Sata, Joji Kitayama

**Affiliations:** 1 Department of Gastrointestinal Surgery, Jichi Medical University, Yakushiji 3311-1, Shimotsuke, Tochigi, 329-0498 Japan; 2 Department of Clinical Oncology, Jichi Medical University, Shimotsuke, Japan; 3 Department of Plastic Surgery, Jichi Medical University, Shimotsuke, Japan

**Keywords:** Abscopal effect, Adenosine, CD73, Radiation, Rectal cancer

## Abstract

**Background:**

Anti-tumor effects of radiation therapy (RT) largely depend on host immune function. Adenosine with its strong immunosuppressive properties is an important immune checkpoint molecule.

**Method:**

We examined how intra-tumoral adenosine levels modify anti-tumor effects of RT in a murine model using an anti-CD73 antibody which blocks the rate-limiting enzyme to produce extracellular adenosine. We also evaluated CD73 expression in irradiated human rectal cancer tissue.

**Results:**

LuM-1, a highly metastatic murine colon cancer, expresses CD73 with significantly enhanced expression after RT. Subcutaneous (sc) transfer of LuM-1 in Balb/c mice developed macroscopic sc tumors and microscopic pulmonary metastases within 2 weeks. Adenosine levels in the sc tumor were increased after RT. Selective RT (4Gyx3) suppressed the growth of the irradiated sc tumor, but did not affect the growth of lung metastases which were shielded from RT. Intraperitoneal administration of anti-CD73 antibody (200 μg × 6) alone did not produce antitumor effects. However, when combined with RT in the same protocol, anti-CD73 antibody further delayed the growth of sc tumors and suppressed the development of lung metastases presumably through abscopal effects. Splenocytes derived from RT+ CD73 antibody treated mice showed enhanced IFN-γ production and cytotoxicity against LuM-1 compared to controls. Immunohistochemical studies of irradiated human rectal cancer showed that high expression of CD73 in remnant tumor cells and/or stroma is significantly associated with worse outcome.

**Conclusion:**

These results suggest that adenosine plays an important role in the anti-tumor effects mediated by RT and that CD73/adenosine axis blockade may enhance the anti-tumor effect of RT, and improve the outcomes of patients with locally advanced rectal cancer.

## Background

Neoadjuvant radiation therapy (RT) can down-stage locally advanced rectal cancer (RC) which results in a lower rate of postoperative local recurrences [1, 2] and is now considered standard treatment for locally advanced RC worldwide. Recent studies have shown that combined RT and fluorouracil-based chemotherapy results in a further improved locoregional control rate without a significant increase in side effects [3, 4]. More recently, other radiosensitizers have been used in clinical trials to improve the efficacy and tolerability of RT.

Although direct cytotoxicity via DNA double-strand breaks or the induction of apoptosis have been considered to be the main mechanisms, a reduction in tumor size is also strongly dependent on host immune responses [5, 6]. In general, it is believed that RT induces transient immunosuppression. However, multiple reports have suggested that tumor cells which are dead or dying due to RT can present tumor-associated antigens to host immune cells and thereby evoke innate and adaptive immune responses [7, 8]. This not only increases the cytotoxic effect on tumor cells directly exposed to RT but also causes regression of tumors outside the irradiated field, the so-called “abscopal effect” [9, 10].

With the recent remarkable progress in the understanding of immune checkpoint molecules, many studies have been performed to evaluate the efficacy combined RT and immunotherapy. Pre-clinical studies have demonstrated that anti-tumor effects of RT are further enhanced by the concurrent administration of antibodies to CTLA-4 and PD-1 [10–12]. Clinical trials have suggested synergistic effects between RT and recently approved antibody preparations against PD-1 and CTLA-4 [13, 14]. In other clinical studies, however, benefits from combined modality therapy have not been confirmed [15, 16]. Therefore, the optimal dose or fractionation of RT as well the nature of agents to optimize the response to RT remain to be elucidated.

Adenosine is an important endogenous regulator of innate and the adaptive immune system. Adenosine strongly suppresses immune cells mainly through the A2A receptor and plays a critical role in the maintenance of homeostasis in various tissues [17, 18]. Adenosine is either released from stressed or injured cells or generated from extracellular adenine nucleotides (ATP (adenosine triphosphate), ADP (adenosine diphosphate) and AMP (adenosine monophosphate)) by the concerted action of the ectoenzymes ectoapyrase (CD39) and 5′ectonucleotidase (CD73). CD39 catalyzes the hydrolysis of ATP/ADP to AMP and CD73 converts AMP to adenosine, and CD73 mediated conversion is considered to be the rate-limiting enzyme in adenosine production [19, 20]. ATP is one of the damage-associated molecular patterns (DAMPs) that function as immunostimulatory signals [21]. Since adenosine, in contrast, exerts strong immunosuppressive functions, balancing ATP and adenosine is believed to be crucial for the local immune response [18, 22].

Malignant cells often express CD73 and high CD73 expression in tumor tissue has been linked to poor clinical outcomes [23] [24, 25], suggesting that adenosine produced by the enzymatic activity of CD73 promotes metastases and survival of tumor cells through immunosuppression. In fact, many pre-clinical studies have shown that inhibition of the CD73/adenosine axis can inhibit tumor progression [26–28]. Those results suggest that modulation of adenosine levels in the tumor microenvironment can be a novel therapeutic strategy to suppress tumor growth [29, 30]. In this study, we examined the role of the CD73/adenosine axis on the tumor response to local RT using a murine model of spontaneous lung metastases and tissue samples from patients with RC.

## Methods

### Reagents and mAbs

Anti-mouse CD73 mAb (clone TY/23) and Rat IgG2a isotype control (clone 2A3) were purchased from BioX-Cell (West Lebanon, NH, USA). Anti-mouse mAbs for flowcytometric analysis were used as described here. FITC-conjugated anti-CD8a (53–6.7), anti-CD11b (M1/70), and PE-conjugated anti-CD4 (GK1.5), anti-CD39 (Duha59), anti-CD45 (30-F11), anti-Ly-6G/Gr-1 (RB6-8C5), anti-IFN-γ (XMG1.2), and APC-conjugated anti-CD3 (17A2), anti-CD45 (30-F11), anti-CD73 (TY/11.8), and BV421 conjugated anti-CD4 (GK1.5), anti-CD45 (30-F11) and mouse recombinant Interleukin-2 (r-IL-2) were purchased from BioLegend (San Diego, CA, USA). FcR blocking reagent was obtained from Miltenyi Biotec GmBH (Bergisch Gladbach, Germany). 7-AAD (7-Aminoactinomycin D) and FVS780 were purchased from Thermo-Fisher Scientific (Waltham, MA, USA) and BD Biosciences (Franklin Lakes, NJ, USA), respectively.

### Cell culture and animal experiments

LuM-1, a highly metastatic sub-clone of murine colon cancer, colon26 [31] was kindly obtained from Dr. Oguri, Aichi Cancer Center, Japan., and maintained in DMEM supplemented with 10% FCS, 100 U/mL penicillin and 100 μg/mL streptomycin (Sigma-Aldrich, St. Louis, MO, USA). After achieving > 80% confluence, cells were removed by treatment with 0.25% (w/v) trypsin solution containing 0.04% (w/v) EDTA, and then used. The cultured cells were tested by the *Mycoplasma* Detection Kit (R&D Systems, Minneapolis, MN, USA) in every 3 months and cells with passages 3 to 5 were used for experiments. Female Balb/c mice age 7–8 weeks were purchased from CLEA Japan (Shizuoka, Japan) and housed in specific pathogen-free (SPF) conditions.

LuM-1 cells (1 × 10^6^) were subcutaneously injected in the right flank of 8–9 weeks-old female Balb/c mice. When the primary tumors reached a volume of 100 to 150 mm^3^ at day12, the mice were divided into groups with each group containing 5 ~ 8 mice to enable the statistical evaluation. Local RT was delivered using MX-160 Labo (mediXtec, Chiba, Japan), as described previously [32]. In short, anesthetized mice were held in the decubitus position, and X-ray irradiation was delivered only to the subcutaneous (sc) tumor with the remainder of the body of the mouse including the lung covered with a 5 mm lead plate. We confirmed the effectiveness of shielding by this method. Mice received 3 fractions of 4 Gy every other day (days 12, 14, 16). For immunotherapy, mice received intraperitoneal injection of 200 μg anti-CD73 mAb or Rat IgG2a isotype control on days 12, 14, 16, 19, 22 and 25. All of the mice were sacrificed with cervical dislocation on day 28, and the weight of the sc tumor and number of macroscopic metastatic nodules in lung were evaluated. All the procedures were approved by Animal Care Committee of Jichi Medical University (No 17005–02) and performed according to the Japanese Guidelines for Animal Research.

### Flow cytometry

LuM-1 cells were cultured at a density of 1 × 10^6^ cells/10 cm dish and 10 Gy RT given with the MX-160 Labo and incubated for an additional 24 h. The cells were harvested, incubated with 10 μl FcR blocking reagent for 10 min at 4 °C and incubated with PE-conjugated anti-CD39 and APC-conjugated anti-CD73 mAb for 30 min at a final concentration of 2.5 μg/mL. After washing twice with staining buffer, the cells were incubated with 7-AAD for 15 min on ice and staining intensity analyzed in 7-AAD (−) live cell population using FACS Calibur (BD Bioscience, Franklin Lakes, NJ, USA). For in vivo experiments, LuM-1 (1 × 10^6^) cells were subcutaneously injected in Balb/c mice and treated with 2 fractions of 4 Gy RT as described above. Two days later, tumors were excised and digested using the Tumor Dissociation Kit, mouse (Miltenyi Biotec) with gentleMACs Dissociators (Miltenyi Biotec). After lysis of red blood cells (RBC) with RBC lysis buffer (BioLegend), cells were passed through a 40-μm filter and single cell suspensions stained with APC conjugated anti-CD73 mAb and PE conjugated anti-CD45 mAb, and the expression level of CD73 was analyzed in live tumor cell population defined in 7-AAD (−) CD45 (−) gated area.

T cells producing IFN-γ were identified by intracellular staining. Mice bearing sc LuM-1 tumors received 3 fractions of 4 Gy local RT and an intraperitoneal injection of 200 μg anti-CD73 mAb or Rat IgG2a isotype control every other day (days 12, 14, 16). The mice were sacrificed on day 18, and splenocytes (1 × 10^6^) were cultured in RPMI-1640 + 10% FCS for 6 h in the presence of 1 μl/mL brefeldin A (BioLegend) for the last 2 h. The cells were harvested, fixed, permeabilized using the fixation / permeabilization buffer (BD Bioscience) according to the manufacturer’s instructions and stained with PE-conjugated IFN-γ or isotype control and FITC-conjugated anti-CD8a, APC-conjugated anti-CD3 and BV421-conjugated anti-CD4 mAb as well as FVS780 to exclude dead cells. The ratio of IFN-γ positive cells were calculated in CD3 (+) CD4 (+) or CD3 (+) CD8a (+) gated area using LSRFortessa (BD Bioscience).

### Cytotoxicity

Splenocytes (5 × 10^6^) from treated mice (as described above) were cultured with 1 × 10^6^ irradiated (50 Gy) LuM-1 cells in 24-well tissue culture plates in 2 ml 10% FCS+ RPMI-1640 medium supplemented with 20 ng/ml mouse rIL-2 for 12 days. Activated splenocytes were incubated with LuM-1 at an E/T ratio of 20:1 for 4 h and all cells stained with FITC-conjugated Annexin-V (BioLegend), 7-AAD and APC conjugated anti-CD45 mAb. The ratio of 7-AAD positive dead cells was calculated in the tumor cell population defined in the FSC/SCC and CD45 (−) gated areas.

### Quantification of adenosine levels in tumor tissue

Quantitative analysis of adenosine, AMP and inosine was performed using an LC-MS system consisting of Nexera X2, LCMS-8060 and a LC/MS/MS Method Package for Primary Metabolite Version 2 (Shimadzu Corp, Kyoto, Japan) as described previously [33]. In brief, sc tumors irradiated as described above (4Gyx2) were resected at 12, 24 and 48 h after treatment and dissociated. Chromatographic separation was performed at 40 °C on a Discovery® HS F5–3 column, 150 × 2.1 mm, 3 μm, (Sigma-Aldrich) with a flow rate of 0.25 mL/min. A gradient elution of mobile phase A consisting of 0.1% of formic acid in water and mobile phase B consisting of 0.1% of formic acid in acetonitrile. The mobile phase B concentration was programmed as follows: 0% (0 min) – 0% (2.0 min) – 25% (5.0 min) – 35% (11 min) – 95% (15 min) – 95% (20 min) – 0% (20.1 min). Nitrogen gas was used as the nebulizer gas with drying gases at flow rates of 3.0 and 10 L/min, respectively. Dry air for the heating gas was at 10 L/min. Collision-induced dissociation (CID) was conducted by argon gas (purity, > 99.9995%). Interface, heat block, and desolvationline temperatures were set at 300, 400, and 250 °C, respectively. Multiple reaction monitoring (MRM) transitions for adenosine, AMP, and inosine were m/z 268.1 > 136.05, m/z 384.0 > 136.05 and m/z 269.1 > 137.05, respectively, in positive ion mode. MRM transition for 2-MES was m/z 194.0 > 80.15 in negative ion mode. The polarity switching time of the instrument was 5 ms (10 ms/cycle).

### Immunohistochemistry of patient samples

Between 2008 and 2015, 64 patients with locally advanced RC received neoadjuvant chemoradiotherapy (CRT) in the Department of Surgery, Division of Gastroenterological General and Transplant Surgery, Jichi Medical University Hospital. Patients were treated with long-course RT (a dose of 50.4 Gy in 25 fractions) using 4-field box techniques. Some patients received concurrent chemotherapy with oral UFT or S1. Radical resections were performed at 8–10 weeks after the end of CRT. The excised tumors were immediately fixed in 10% buffered formalin, and consecutive formalin-fixed paraffin-embedded 4-μm sections prepared for immunohistochemical evaluation.

After treatment with xylene and ethanol and washing with phosphate-buffered saline (PBS), tumor specimens were subjected to heat-induced antigen retrieval in citrate buffer (Muto Pure Chemicals Co., Ltd., Tokyo, Japan) followed by endogenous peroxidase blocking by Peroxidase-Blocking solution (DAKO, Santa Clara, CA, USA). The tissues were washed with PBS and incubated with 5% bovine serum albumin for 30 min to block nonspecific antibody binding. The slides were then incubated overnight at 4 °C with monoclonal antibodies against CD73 (D7F9A, Rabbit IgG, Cell Signaling Technology, Danvers, MA, USA) at a dilution of 1:200 in humid chambers overnight at 4 °C. After three 5-min washes with PBS, sections were incubated with anti-rabbit secondary antibody conjugated with peroxidase for 30 min at room temperature. After washing, the enzyme substrate 3,30-diaminobenzidine (Dako REAL EnVision Detection System, DAKO) was used for visualization and counterstained with Meyer’s hematoxylin.

Staining intensities in remnant tumor cells or stroma were independently scored from 0 to 3 (Fig. S6) by two different evaluators who were unaware of the clinical findings, and the cases were divided into high (score = 2 or > 2) and low (score < 2) expression groups by the mean score of the two evaluators. This study protocol was approved by the institutional IRB of Jichi Medical University (Rin A17–164) and conducted in accordance with the guiding principles of the Declaration of Helsinki. Written informed consent was obtained from all participants.

### Statistical analysis

Data are presented as the means ± SEM or median (min-max). Statistical differences were analyzed by student-t-test, the Mann-Whitney test, one-way ANOVA with post hoc test with Tukey’s or Dunnett’s procedure, the χ2-test or Fisher’s exact test and *p* values less than 0.05 were considered significant. Recurrence free survival (RFS) and overall survival (OS) rates were calculated using the Kaplan-Meier method and differences were evaluated using the log-rank test. Uni- and multivariate analyses were performed using the Cox proportional hazard model to evaluate the predictors of prognosis. Statistical analysis was performed using GraphPad Prism 7 software (GraphPad Software Inc., San Diego, CA, USA) or IBM SPSS Statistics 21 (IBM, Chicago, IL, USA).

## Results

### RT enhances the expression of CD73 by LuM-1 cells and increases adenosine levels in subcutaneous tumors

**Fig. 1:**
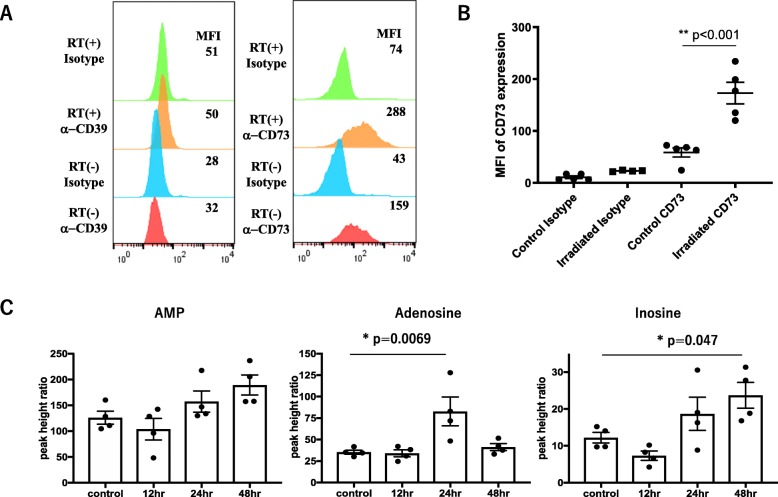
Radiation therapy (RT) enhances the membrane expression of CD73 and increases adenosine levels in subcutaneous (sc) LuM-1 tumors. **a** Cultured LuM-1 cells were treated with or without 10 Gy RT using the MX-160 Labo (mediXtec), and incubated for an additional 24 h. The cells were stained with mAbs to CD39 (left) or CD73 (right) and mean fluorescein intensities (MFI) in the 7AAD (−) live cell population were examined by FACS. Data show a representative FACS profile in 5 different experiments. **b** Two fractions of 4 Gy RT were delivered selectively to sc tumors of LuM-1 with the remainder of Balb/c mice shielded by a lead plate. Two days later, tumors were resected and single cell suspensions obtained using a Tumor Dissociation Kit (Miltenyi Biotec). The cells were stained with mAbs to CD73 and CD45, and MFI for CD73 were analyzed in live tumor cells defined by 7AAD (−) CD45(−) gated area. *P* value was calculated with one-way ANOVA followed by Tukey test. **c** Two fractions of 4 Gy RT were delivered to sc tumors as described above, which were removed at 12, 24 and 48 h after RT. Levels of AMP (adenosine monophosphate), adenosine, and inosine levels in those samples were measured with the LC-MS system (Shimadzu Corp) as described in material and methods. The Y axis shows the height ratio between the 2-morpholino-ethanesulfonic acid (2-ME) as an internal standard and the target molecules. *P* value was calculated with one-way ANOVA followed by Dunnett’s test and * showed *p* < 0.05

The expression of CD39 and CD73 in cultured LuM-1cells was examined with flow cytometry. CD39 was scarcely expressed on LuM-1 cells and not changed after RT (Fig. 1a, left panel). In comparison, LuM-1 cells positively expressed CD73 and its expression level was further enhanced 24 h after treatment with 10 Gy RT (Fig. 1a, right panel and Fig. S1A). RT (4Gy × 2) was given to sc LuM-1 tumors implanted in syngeneic mice, and CD73 expression in the LuM-1 cells recovered from resected tumors were evaluated by mean fluorescein intensity (MFI) in CD45 (−) tumor cells. Consistent with the in vitro results, CD73 expression level in LuM-1 cells was significantly enhanced compared with non-irradiated controls (Fig. 1b). MFI in CD45 (+) cells did not show significant difference (Fig. S1B).

The levels of adenosine, as well as its precursor, AMP and its metabolite, inosine, in irradiated sc tumors were examined using an LC-MS system. As evaluated by the peak height ratio against the internal standard, adenosine levels in tumors were significantly increased at 24 h after 2 cycles of RT, and inosine levels were significantly increased at 48 h after RT (Fig. 1c).

### Anti-CD73 mAb combined with RT suppresses non-irradiated lung metastases as well as irradiated tumor

**Fig. 2:**
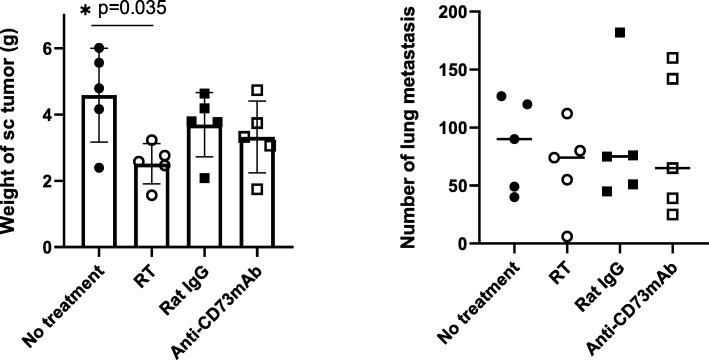
Anti-tumor effects of radiation therapy (RT) or anti-CD73 antibody used alone. Tumor bearing mice received local RT to subcutaneous (sc) tumors (3 fractions of 4 Gy) as described above on days 12, 14, 16 and intraperitoneal injection of 200 μg anti-CD73 mAb or Rat IgG2a isotype control at days 12, 14, 16, 19, 22 and 25. All of the mice were sacrificed on day 28, and the weight of sc tumors and number of macroscopic metastatic nodules in the lungs evaluated. *P* value was calculated with one-way ANOVA followed by Tukey test and * showed *p* < 0.05

In a preliminary experiment, we confirmed that all mice developed sc tumors with micrometastases in both lungs at 12 days after subcutaneously injection of LuM-1 cells, although all mice were healthy with apparent sc tumor. When local RT (4Gy × 3) was delivered selectively to sc tumors after 12 days, the weight of the sc tumor at day 28 was significantly reduced (2.5 ± 0.61 g vs 4.8 ± 0.61 g, *p* < 0.05, *n* = 5), while the number of lung metastases was not altered. Treatment with anti-CD73 mAb alone did not show significant difference from isotype control for the sc tumor or the lung metastases (Fig. 2).

**Fig. 3:**
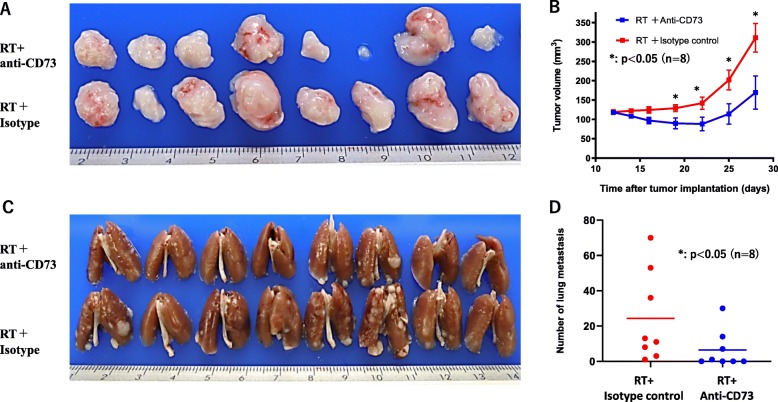
Anti-tumor effects of anti-CD73 antibody combined with radiation therapy (RT). Tumor bearing mice received local RT together with immunotherapy using the same protocol shown in Fig. 2. The growth of subcutaneous (sc) tumors was evaluated by their volume calculated by length×width^2^/2 (**b**). All mice were sacrificed on day 28, and the volume of sc tumor (**a**) as well as the number of macroscopic metastatic nodules (**c**, **d**) in the lungs counted. *P* value was calculated with the Mann-Whitney test and * showed *p* < 0.05

However, when RT was delivered to sc tumor together with administration of anti-CD73 mAb or isotype control, the growth of sc tumor was significantly delayed in mice treated with anti-CD73 mAb (*p* < 0.05, at day 18 and later) and tumor volume at day 28 was reduced to about 50% (Fig. 3a, b). Moreover, the number of lung metastases was significantly reduced in anti-CD73 mAb treated mice (1, 0 ~ 30 vs 12, 1 ~ 70, *p* = 0.04, *n* = 8). No metastases were observed in 4/8 mice treated with RT+ anti-CD73 mAb, although metastases developed in all mice in the control group (Fig. 3c, d). Same trend was observed in 2 different experiments with 2 cycles of RT although the differences were not statistically significant (Fig. S2).

### Anti-CD73 mAb combined with RT enhances the systemic immune response

**Fig. 4:**
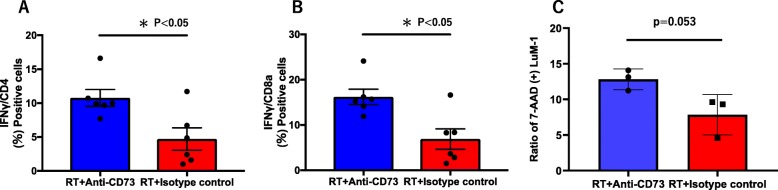
Effects of anti-CD73 antibody on splenocytes of irradiated tumor bearing mice. CD73 mAb enhances IFN-γ production and cytotoxicity of splenocytes from irradiated mice. **a, b** Tumor bearing mice received 3 fractions of 4 Gy local radiation therapy (RT) together with intraperitoneal injection of 200 μg anti-CD73 mAb or Rat IgG2a isotype control on days 12, 14, 16, and sacrificed at day 18. The splenocytes were cultured in RPMI-1640 + 10% FCS in the presence of brefeldin A and then fixed, permeabilized and stained with PE-conjugated IFN-γ or isotype control and APC-conjugated anti-CD3 and BV421-conjugated anti-CD4 mAb and FITC-conjugated anti-CD8 mAb as well as FVS780 for dead cell exclusion. The ratio of IFN-γ positive cells were calculated in CD3 (+) CD4 (+) or CD3 (+) CD8a (+) gated area. **c** The splenocytes were cultured with irradiated LuM1 in 2 ml 10% FCS+ RPMI-1640 medium supplemented with 20 ng/ml mouse recombinant IL-2 for 12 days, and then incubated with LuM-1 cells at an E/T ratio of 20:1. After 4 h incubation, all cells were stained with FITC-conjugated Annexin-V, 7-AAD and APC-conjugated anti-CD45 mAb, and ratios of 7-AAD positive dead cells calculated in LuM-1 population defined in FSC/SCC and CD45 (−) gated area. *P* value was calculated with the Mann-Whitney test and * showed *p* < 0.05

We then examined lymphocyte populations in the spleens and infiltrating lymphocytes in sc tumor of tumor-bearing mice. The ratios of CD4 (+) or CD8a (+) T cells and CD11b (+) Gr-1(+) myeloid derived suppressor cells were not altered comparing the anti-CD73 mAb treated and isotype control groups (Fig. S3, S4). However, as shown in Figs. 4a and b, intracellular staining showed that IFN-γ producing cells were significantly increased in CD4 (+) and CD8a (+) T cells in anti-CD73 mAb treated mice (CD4; 10.8 ± 1.2% vs 4.7 ± 1.6%, *p* < 0.05, *n* = 6: CD8a; 16.2 ± 1.7% vs 6.9 ± 2.3%, *p* < 0.05, n = 6). Moreover, infiltrating lymphocytes in sc tumor showed the same trend with statistical significance in CD4 (+) population (Fig. S5).

After co-culture with irradiated LuM-1 cells and rIL-2 for 12 days, splenocytes in RT and anti-CD73 mAb treated mice tended to show increased cytotoxicity against LuM-1 with marginal significance (12.8 ± 1.5% vs 7.8 ± 2.8% at E/T ratio = 20, *p* = 0.053, *n* = 3) (Fig. 4c).

### Expression of CD73 in tumor cells or stroma correlates with the outcomes of patients who received neoadjuvant RT

**Fig. 5:**
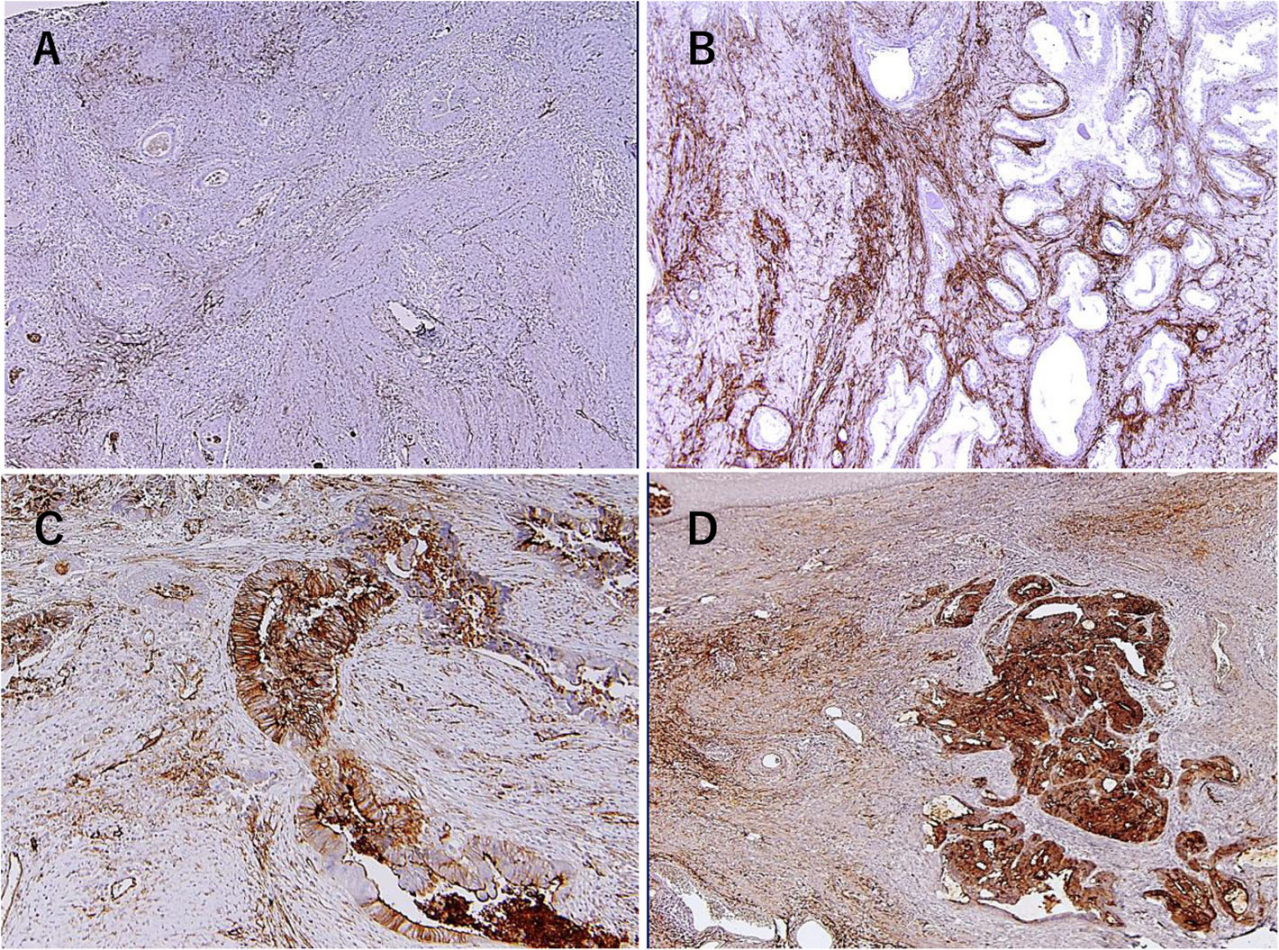
CD73 expression in rectal cancer tissue after chemoradiation therapy. Formalin-fixed paraffin-embedded 4-μm sections were immune stained with polyclonal Ab to human CD73 using REAL EnVision Detection System (DAKO) as described in Methods. The staining intensities were separately evaluated in remnant tumor cells or stroma and divided into high and low expression groups. Four representative cases, **a** remnant tumor cells low, stroma low **b** remnant tumor cells low, stroma high **c** remnant tumor cells high, stroma low **d** remnant tumor cells high, stroma high were shown

**Fig. 6:**
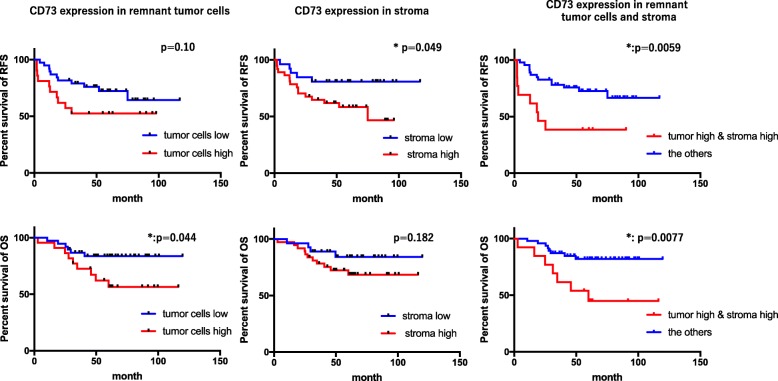
Impact of CD73 expression on outcome of 64 patients who received preoperative radiation therapy for locally advanced rectal cancer. Patients were divided into CD73 high and low expression groups either in remnant tumor cells (left panels) or stroma (middle panels), as well as high in both areas or others (right panels), and recurrence free survival (RFS; upper panels) and overall survival (OS; lower panels) were compared with Kaplan-Meier method. *P* values were calculated by the log-rank test and * showed *p* < 0.05

The expression of CD73 in 64 surgically resected specimens from patients with RC who had received neoadjuvant CRT was immunohistochemically evaluated. The outcomes of these patients was evaluated with regard to CD73 expression. As shown in Fig. 5, remnant cancer cells and stroma were stained positive for CD73 and the staining pattern was highly variable among the patients. Therefore, we separately evaluated the staining intensity in remnant tumor cells and stroma (Fig. S6) and divided these into high and low expression groups (Fig. 5 and Table 1). The CD73 expression level did not show significant correlation with clinical or pathological findings including pathological response (Table 1). However, recurrence in distant sites tended to be observed frequently in patients with higher-expressing CD73 tumors (Table 1). Accordingly, patients with tumors showing high CD73 expression either in remnant tumor cells or stroma tended to have shorterRFS and OS compared to patients with low CD73 expression (Fig. 6). Especially, 13 patients with tumors that highly express CD73 both in remnant tumor cells and stroma showed markedly worse outcomes compared to the other 51 patients (*p* = 0.0059) with mean RFS of 22 months (Fig. 6 right panels). In the univariate analysis, high CD73 expression both in remnant tumor cells and stroma was significantly associated with worse prognosis (Table S1). In the multivariate analysis, high CD73 expression both in remnant tumor cells and stroma remained an independent predictor of RFS and OS (Table S1).

**Table 1:** CD73 expression levels and clinical and pathological findings of 64 patients with rectal cancer treated with neoadjuvant radiation therapy

Variable	Tumor cells (64)				Stroma (64)		
	Low (38)	High (22)	Unknown (4)	***p***-value	Low (27)	High (37)	***p***-value
**Age**	**63 (36–78)**	**59 (42–79)**	**66 (63–68)**	**0.13**	**61 (36–74)**	**62 (44–79)**	**0.8**
**Gender**							
**M**	**29**	**16**	**3**	**0.77**	**19**	**29**	**0.56**
**F**	**9**	**6**	**1**		**8**	**8**	
**Location**							
**Rab**	**2**	**1**		**0.69**	**2**	**1**	**0.48**
**Rb**	**36**	**21**			**25**	**36**	
**Histology**							
**Differentiated**	**32**	**20**		**0.7**	**22**	**31**	**0.46**
**Undifferentiated**	**6**	**2**			**2**	**6**	
**Lymphatic invasion**							
**Absent**	**24**	**11**		**0.42**	**17**	**22**	**0.8**
**Present**	**14**	**11**			**10**	**15**	
**Venous invasion**							
**Absent**	**13**	**9**		**0.78**	**10**	**16**	**0.8**
**Present**	**25**	**13**			**17**	**21**	
**Tumor Stage**							
**t0/t1**	**4**	**0**	**4**	**0.48**	**5**	**3**	**0.35**
**t2**	**9**	**5**			**7**	**7**	
**t3**	**23**	**14**			**12**	**25**	
**t4**	**2**	**3**			**3**	**2**	
**N stage**							
**n0**	**23**	**15**		**0.59**	**17**	**25**	**0.79**
**n1<**	**15**	**7**			**10**	**12**	
**Pathological response**							
**grade 0/1**	**25**	**17**		**0.29**	**18**	**24**	**0.63**
**grade 2**	**13**	**5**	**1**		**7**	**12**	
**grade 3**	**0**	**0**	**3**		**2**	**1**	
**Combined Chemotherapy**							
**Not given**	**3**	**4**	**0**	**0.31**	**4**	**3**	**0.2**
**Given**	**35**	**18**	**4**		**23**	**34**	
**Adjuvant chemotherapy**							
**Given**	**16**	**5**		**0.13**	**9**	**12**	**0.99**
**Not Given**	**22**	**17**	**4**		**18**	**25**	
**Recurrence**							
**Present**	**11**	**11**		**0.16**	**6**	**16**	**0.11**
**Absent**	**27**	**11**	**4**		**21**	**21**	

CD73 expression in tumor cells cannot be appropriately evaluated in 1 patient with a grade 2 response due to few remaining tumor cells as well as in 3 patients with grade 3 responses (pathological complete response). Statistical significance of the differences was evaluated by student-t-test, the Mann-Whitney test, the χ^2^-test and Fisher’s exact test

## Discussion

RT has been widely used for the treatment of solid tumors either with curative intent or as palliative treatment. Recent clinical [13, 14] as well as pre-clinical [10–12] studies have suggested that tumor responses to RT are significantly enhanced by combination with immune checkpoint blockade. Adenosine has a strong immunosuppressive property and is now considered as an important “metabolic immune checkpoint molecule” [22, 34]. Inhibition of the CD73/adenosine axis attracts attention as a novel form of immunotherapy that could be combined with RT [35, 36]. However, it is unclear how the modulation of adenosine levels affect the outcome of RT.

In this study, we found that CD73 is significantly expressed in a highly metastatic clone of colon26, LuM-1, and was further upregulated by irradiation both in vitro and in vivo. Previous studies have shown that CD73 gene expression is enhanced by hypoxia [37] and proinflammatory cytokines [38] which are often associated with RT. RT has been shown to upregulate CD73 expression in esophageal [39] and bladder cancer [40] cells as well as immune cells [41], which is consistent with the present results. Since after RT large amounts of adenosine precursors are expected to be released into the extracellular space from damaged cells, it is possible that upregulation of CD73 causes large amounts of adenosine to accumulate in irradiated tumor tissue.

Accurate quantification of tissue adenosine levels is challenging because of its low molecular weight, high polarity and short half-life due to enzymatic degradation [42]. Previous studies using reversed phase high pressure liquid chromatography showed that extracellular adenosine levels in solid tumors were 50–100 μM, which is higher than those in normal tissue and enough to suppress local antitumor immune responses [43, 44]. In this study, we used the LC-MS method with superior sensitivity and selectivity compared with conventional liquid chromatography [45], and found that adenosine levels in sc LuM-1 tumors are significantly elevated 24 h after RT. To the best of our knowledge, this is the first report to directly evaluate changes in adenosine levels in irradiated tumors. Levels of inosine, a stable metabolite of adenosine, were increased at a later time. These results suggest that adenosine levels in the microenvironment of irradiated tumors are maintained at considerably high levels, at least for hours, which may attenuate the anti-tumor immune response elicited by RT.

In this study, RT (4Gy × 3) delayed the growth of sc LuM-1 tumors while anti-CD73 antibody did not show anti-tumor effects when used alone. However, when combined with RT, antibody administration further suppressed the growth of irradiated tumors compared with tumor growth in isotype control treated mice. Anti-CD73 antibody significantly reduced the number of metastases in the lungs, which had not been irradiated. No metastases were observed in the lungs of 50% of mice treated with anti-CD73 together with RT. Since microscopic metastases already existed in the lungs at the time of treatment, it is suggested that the combination of RT and anti-CD73 antibody evokes a systemic immune response which eliminated tumor cells in the lung. Splenocytes from mice treated with RT and anti-CD73 antibody had an increased ability to produce IFN-γ and enhanced cytotoxicity against autologous LuM-1 in vitro. These results suggest that anti-CD73 antibody can induce abscopal effects of RT, which might be partially attributed to T cells stimulated by RT-induced tumor-associated antigen.

CD73 is a multifunctional molecule expressed in various cells. Previous studies have shown that CD73 on tumor cells can mediate proliferation and migration apart from its enzymatic activity and that blocking CD73 can suppress tumor growth [46, 47]. In other studies, CD73 has been shown to contribute to the process of angiogenesis via both its enzymatic and non-enzymatic functions [48, 49]. These results suggest that CD73 blockade may suppress the growth of lung metastases through mechanisms unrelated to immunity. In this study, however, it seems to be unlikely because anti-CD73 mAb, when used alone, did not show significant inhibition in lung metastases in vivo. In fact, in vitro proliferation and migration of LuM-1 cells were not affected by CD73 mAb treatment (data not shown).

Immunostaining experiments showed that CD73 was expressed both in remnant tumor cells and/or stroma in surgically resected human RC after CRT. Although the expression pattern differs among patients, high expression of CD73 was associated with poor prognosis with a higher incidence of distant recurrence, which is consistent with previous studies of non-irradiated tumors [23] [24, 25]. This might be partially caused by the concurrent chemotherapy, since chemotherapy induced CD73 expression in epithelial ovarian cancer and CD73 blockade improved the therapeutic efficacy [50]. However, together with the results of the murine experiments, it is suggested that increased adenosine levels, by enhanced CD73 in irradiated tumor tissue, may impair systemic immune responses which might be causally related to the growth of micrometastases in distant organs in human.

There is growing evidence that RT can result in in situ tumor vaccination by exposing tumor specific neoantigens to the host innate immune system, and thus radio- immunotherapy has the possibility of being an effective novel therapy for patients with advanced cancer. However, there are still major challenges to understanding the dual face of RT-induced effects on the immune system. This is the first report to suggest that the anti-tumor response may be reduced by adenosine in irradiated tumor which is restored by functional blockade of CD73. Anti-CD73 mAb has already been used in a phase 1 clinical trial (NCT02503774) [51]. These results of the present study encourage the clinical appreciation of anti-CD73 mAb combined with RT as a promising preoperative treatment for patients with locally advanced RC.

## Conclusion

After local RT, adenosine levels in irradiated tumor is considerably elevated which may reduce the anti-tumor effects mediated by RT through the induction of immunosuppression. The combination with CD73/adenosine axis blockade may enhance local and abscopal effects of RT and improve the outcomes of patients with locally advanced rectal cancer.

## Supplementary information

**Additional file 1: Figure S1.** (A) Cultured LuM-1 cells were treated with or without 10 Gy RT using the MX-160 Labo (mediXtec), and incubated for an additional 24 h. The cells were stained with anti-CD73 mAb and MFI in the 7AAD (−) live cell population were examined by FACS. Data in 5 different experiments were expressed. (B) Two fractions of 4 Gy RT were delivered selectively to sc tumors of LuM-1 with the remainder of Balb/c mice shielded by a lead plate. Two days later, tumors were resected and single cell suspensions obtained using a Tumor Dissociation Kit. The cells were stained with mAbs to CD73 and CD45, and MFI for CD73 were analyzed in live tumor cells defined by 7AAD (−) CD45(+) gated area. *P* value was calculated with one-way ANOVA followed by Tukey test. **Figure S2.** Tumor bearing mice received local RT to sc tumors (2 fractions of 4 Gy) on days 14, 16 and/or an intraperitoneal injection of 200 μg anti-CD73 mAb or Rat IgG2a isotype control at days 16, 19, 22 and 25. The growth of sc tumors and the number of lung metastases were evaluated by their volume calculated by length×width^2^/2. *P* value were calculated with ANOVA with Tukey’s test. **Figure S3.** Tumor bearing mice treated as Fig. 3 and sacrificed on day 18. Their splenocytes were stained with mAbs to CD3, CD4, CD8a, CD11b and Ly-6G/Gr-1 with FVS780 and positive cells were calculated in FVS780 (−) live cell population. **Figure S4.** Tumor bearing mice treated as Fig. 3 and sacrificed on day 18 and the sc tumors were dissociated with cell dissociation kit and the cells recovered from each tumor were stained with mAbs to CD45, CD3, CD4, CD8a, CD11b and Ly-6G/Gr-1 with FVS780 and positive cells were calculated in FVS780 (−) CD45 (+) live cell population. **Figure S5**. Tumor infiltrating cells were cultured in RPMI-1640 + 10% FCS in the presence of brefeldin A and then fixed, permeabilized and stained with PE-conjugated IFN-γ or isotype control and APC-conjugated anti-CD3 and BV421-conjugated anti-CD4 mAb and FITC-conjugated anti-CD8a mAb as well as FVS780 for dead cell exclusion. The ratio of IFN-γ positive cells were calculated in CD3 (+) CD4 (+) or CD3 (+) CD8a (+) gated area. *P* value was calculated with the Mann-Whitney test. **Figure S6.** Classification of high and low expression of CD73. Staining intensities of CD73 were evaluated in remnant tumor cells or stroma separately by scoring (0, 1+, 2++, 3+++). **Table S1.** Univariate and Multivariate analysis on the correlation between clinicopathological variables and outcomes.

## Data Availability

‘Not applicable’.

## Abbreviation

RT: Radiation therapy
