# Supplementary material for: CD73 blockade enhances the local and abscopal effects of radiotherapy in a murine rectal cancer model
Source: BMC Cancer. 2020 May 12;20:411. doi: 10.1186/s12885-020-06893-3 (PMC7218548; doi:10.1186/s12885-020-06893-3)
Supplement: Supplementary file 1 — Additional file 1: Figure S1. (A) Cultured LuM-1 cells were treated with or without 10 Gy RT using the MX-160 Labo (mediXtec), and incubated for an additional 24 h. The cells were stained with anti-CD73 mAb and MFI in the 7AAD (−) live cell population were examined by FACS. Data in 5 different experiments were expressed. (B) Two fractions of 4 Gy RT were delivered selectively to sc tumors of LuM-1 with the remainder of Balb/c mice shielded by a lead plate. Two days later, tumors were resected and single cell suspensions obtained using a Tumor Dissociation Kit. The cells were stained with mAbs to CD73 and CD45, and MFI for CD73 were analyzed in live tumor cells defined by 7AAD (−) CD45(+) gated area. P value was calculated with one-way ANOVA followed by Tukey test. Figure S2. Tumor bearing mice received local RT to sc tumors (2 fractions of 4 Gy) on days 14, 16 and/or an intraperitoneal injection of 200 μg anti-CD73 mAb or Rat IgG2a isotype control at days 16, 19, 22 and 25. The growth of sc tumors and the number of lung metastases were evaluated by their volume calculated by length×width2/2. P value were calculated with ANOVA with Tukey’s test. Figure S3. Tumor bearing mice treated as Fig. 3 and sacrificed on day 18. Their splenocytes were stained with mAbs to CD3, CD4, CD8a, CD11b and Ly-6G/Gr-1 with FVS780 and positive cells were calculated in FVS780 (−) live cell population. Figure S4. Tumor bearing mice treated as Fig. 3 and sacrificed on day 18 and the sc tumors were dissociated with cell dissociation kit and the cells recovered from each tumor were stained with mAbs to CD45, CD3, CD4, CD8a, CD11b and Ly-6G/Gr-1 with FVS780 and positive cells were calculated in FVS780 (−) CD45 (+) live cell population. Figure S5. Tumor infiltrating cells were cultured in RPMI-1640 + 10% FCS in the presence of brefeldin A and then fixed, permeabilized and stained with PE-conjugated IFN-γ or isotype control and APC-conjugated anti-CD3 and BV421-conjugated anti-CD4 mAb and FITC-co [file 12885_2020_6893_MOESM1_ESM.pptx]

## Slide 1
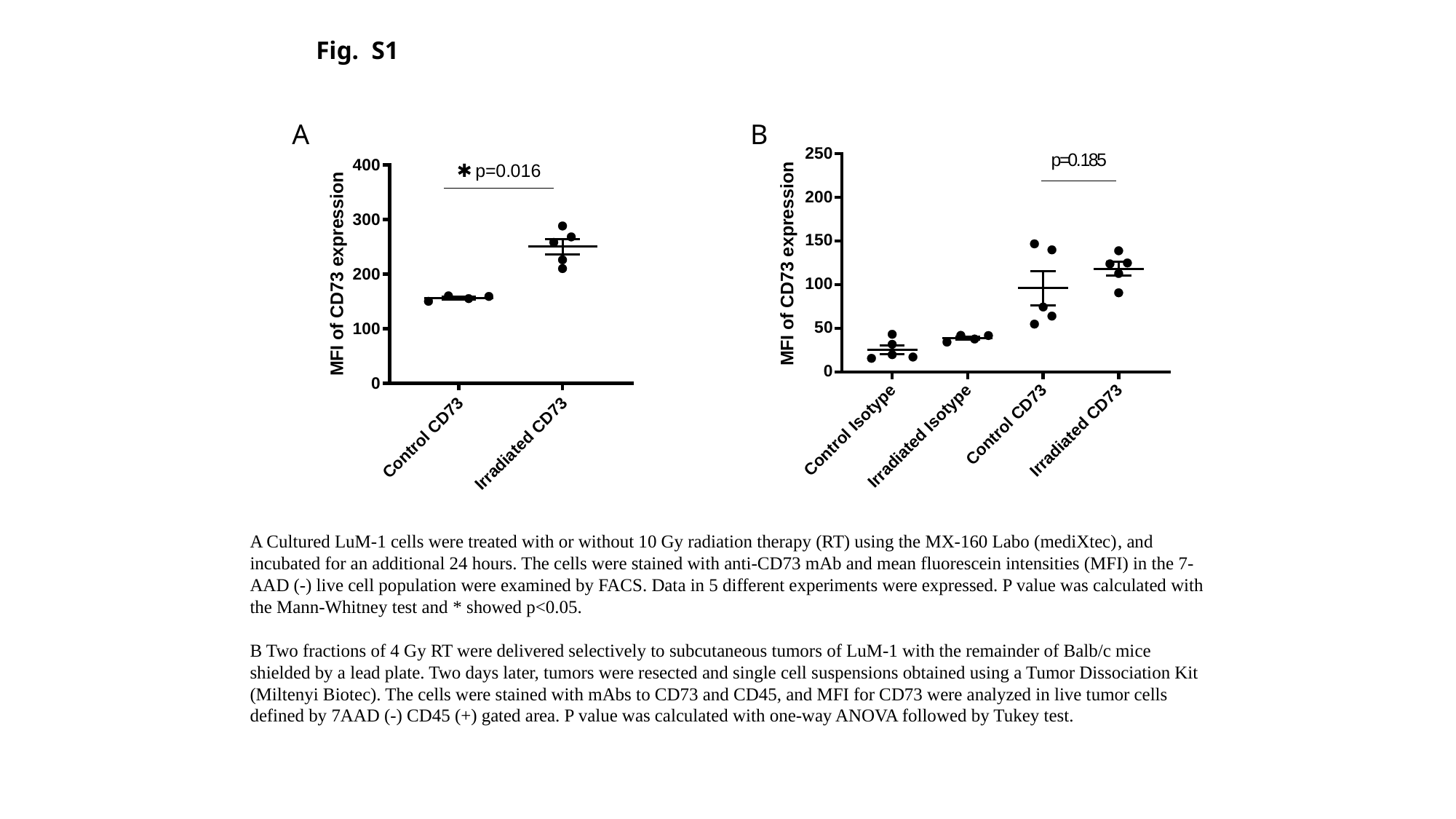

Fig. S1
A
B
A Cultured LuM-1 cells were treated with or without 10 Gy radiation therapy (RT) using the MX-160 Labo (mediXtec), and incubated for an additional 24 hours. The cells were stained with anti-CD73 mAb and mean fluorescein intensities (MFI) in the 7-AAD (-) live cell population were examined by FACS. Data in 5 different experiments were expressed. P value was calculated with the Mann-Whitney test and * showed p<0.05.
B Two fractions of 4 Gy RT were delivered selectively to subcutaneous tumors of LuM-1 with the remainder of Balb/c mice shielded by a lead plate. Two days later, tumors were resected and single cell suspensions obtained using a Tumor Dissociation Kit (Miltenyi Biotec). The cells were stained with mAbs to CD73 and CD45, and MFI for CD73 were analyzed in live tumor cells defined by 7AAD (-) CD45 (+) gated area. P value was calculated with one-way ANOVA followed by Tukey test.

## Slide 2
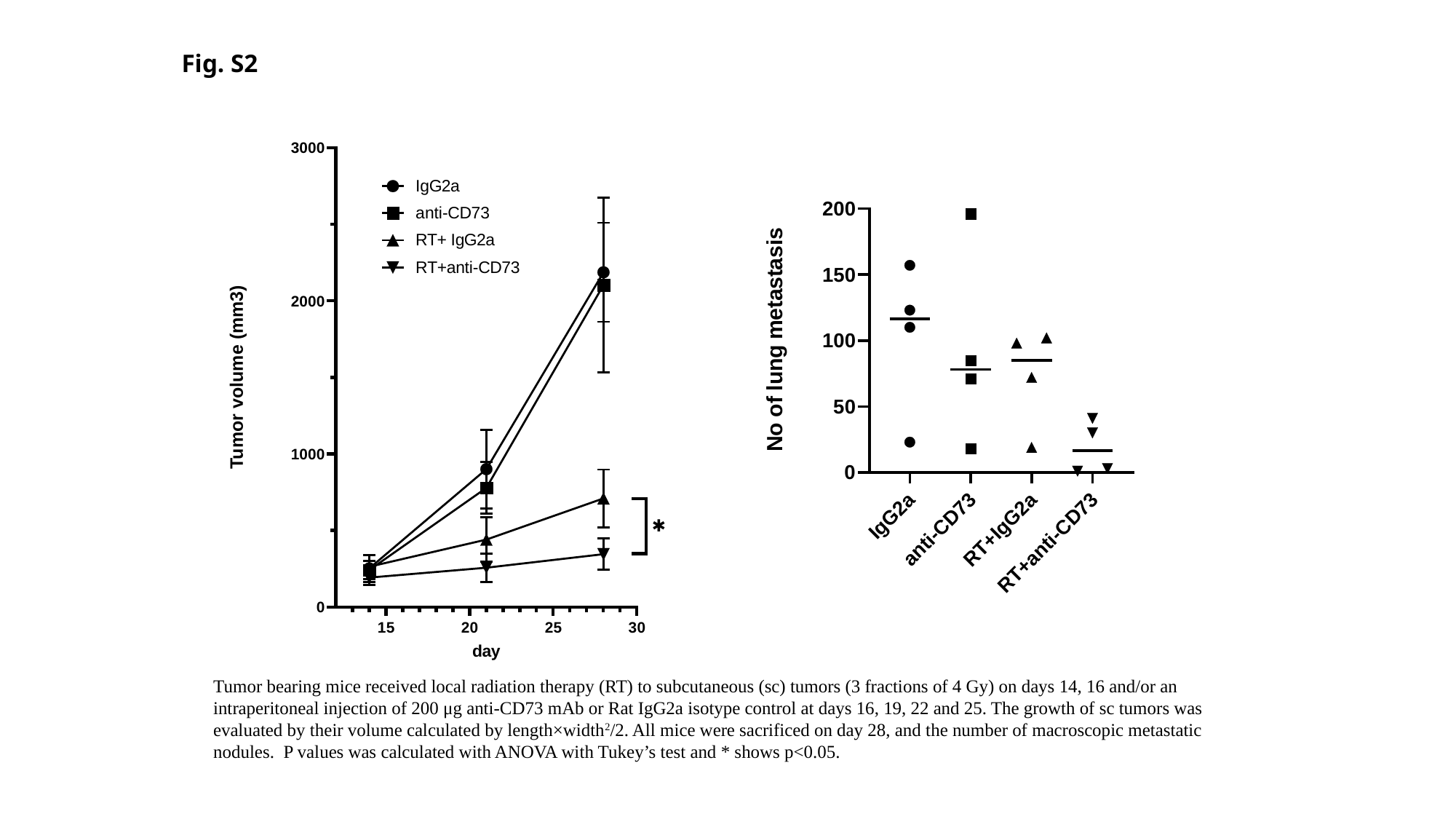

Fig. S2
Tumor volume (mm3)
Tumor bearing mice received local radiation therapy (RT) to subcutaneous (sc) tumors (3 fractions of 4 Gy) on days 14, 16 and/or an intraperitoneal injection of 200 μg anti-CD73 mAb or Rat IgG2a isotype control at days 16, 19, 22 and 25. The growth of sc tumors was evaluated by their volume calculated by length×width2/2. All mice were sacrificed on day 28, and the number of macroscopic metastatic nodules. P values was calculated with ANOVA with Tukey’s test and * shows p<0.05.

## Slide 3
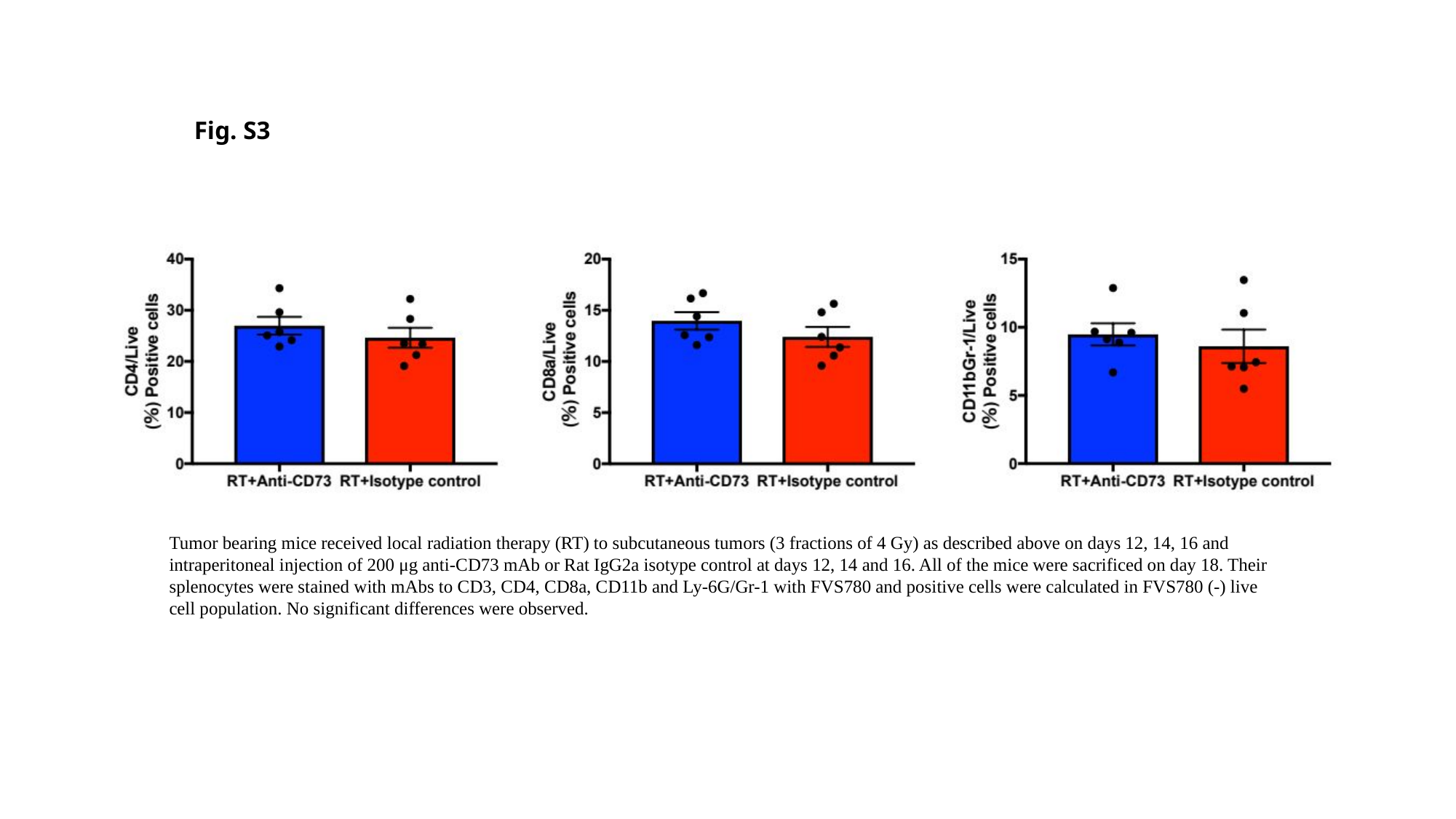

Fig. S3
Tumor bearing mice received local radiation therapy (RT) to subcutaneous tumors (3 fractions of 4 Gy) as described above on days 12, 14, 16 and intraperitoneal injection of 200 μg anti-CD73 mAb or Rat IgG2a isotype control at days 12, 14 and 16. All of the mice were sacrificed on day 18. Their splenocytes were stained with mAbs to CD3, CD4, CD8a, CD11b and Ly-6G/Gr-1 with FVS780 and positive cells were calculated in FVS780 (-) live cell population. No significant differences were observed.

## Slide 4
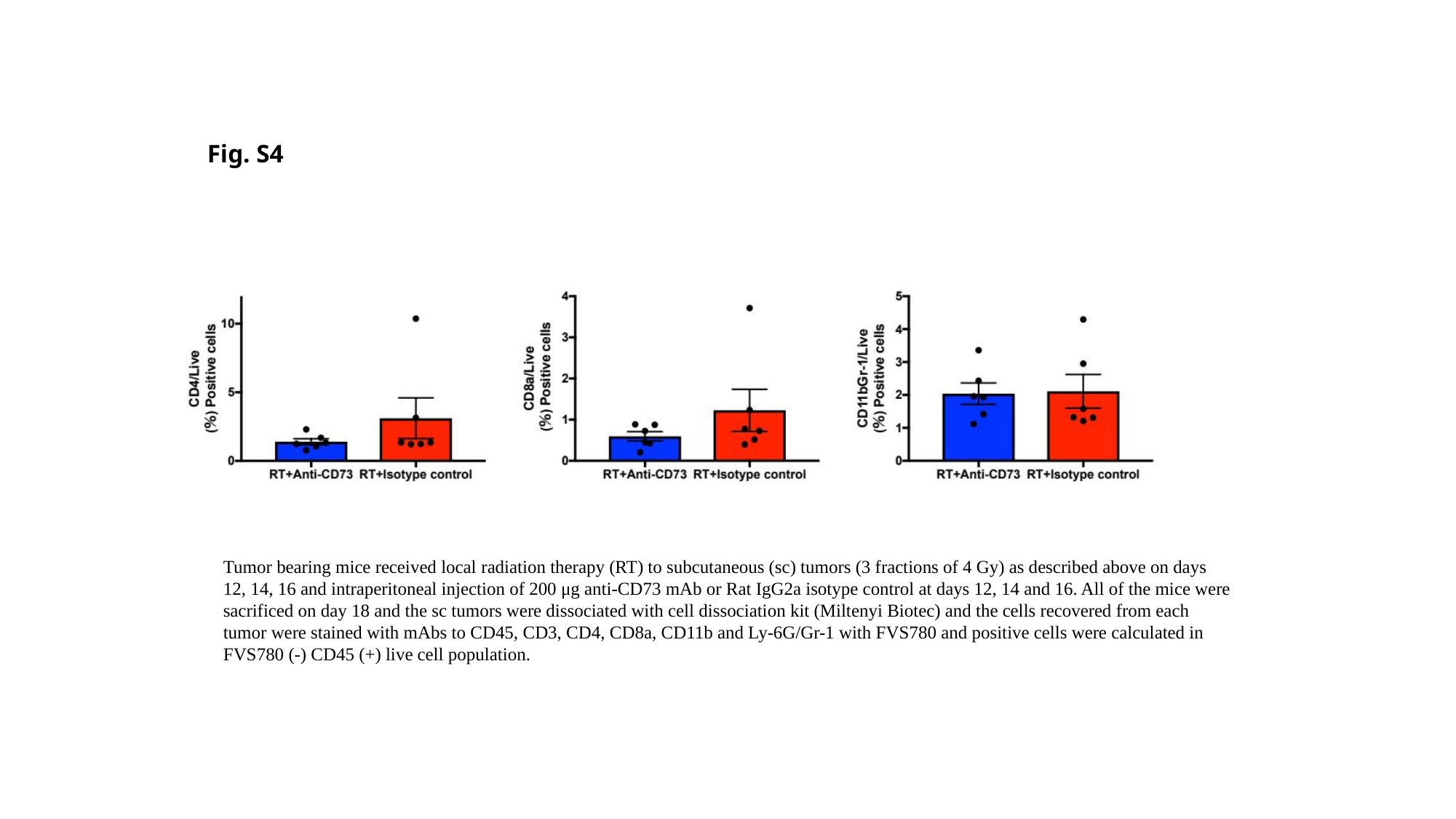

Fig. S4
Tumor bearing mice received local radiation therapy (RT) to subcutaneous (sc) tumors (3 fractions of 4 Gy) as described above on days 12, 14, 16 and intraperitoneal injection of 200 μg anti-CD73 mAb or Rat IgG2a isotype control at days 12, 14 and 16. All of the mice were sacrificed on day 18 and the sc tumors were dissociated with cell dissociation kit (Miltenyi Biotec) and the cells recovered from each tumor were stained with mAbs to CD45, CD3, CD4, CD8a, CD11b and Ly-6G/Gr-1 with FVS780 and positive cells were calculated in FVS780 (-) CD45 (+) live cell population.

## Slide 5
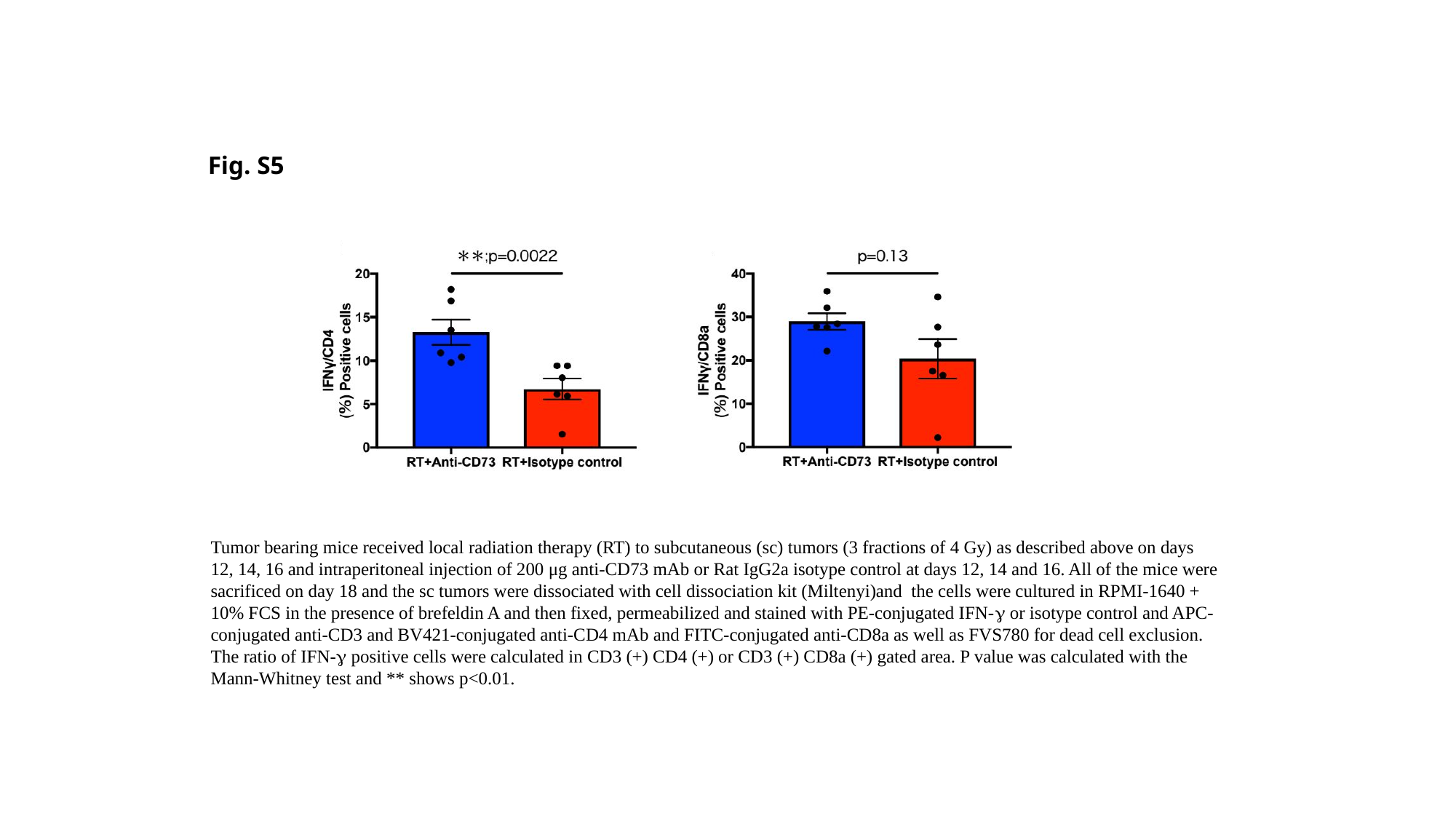

Fig. S5
Tumor bearing mice received local radiation therapy (RT) to subcutaneous (sc) tumors (3 fractions of 4 Gy) as described above on days 12, 14, 16 and intraperitoneal injection of 200 μg anti-CD73 mAb or Rat IgG2a isotype control at days 12, 14 and 16. All of the mice were sacrificed on day 18 and the sc tumors were dissociated with cell dissociation kit (Miltenyi)and the cells were cultured in RPMI-1640 + 10% FCS in the presence of brefeldin A and then fixed, permeabilized and stained with PE-conjugated IFN-g or isotype control and APC-conjugated anti-CD3 and BV421-conjugated anti-CD4 mAb and FITC-conjugated anti-CD8a as well as FVS780 for dead cell exclusion. The ratio of IFN-g positive cells were calculated in CD3 (+) CD4 (+) or CD3 (+) CD8a (+) gated area. P value was calculated with the Mann-Whitney test and ** shows p<0.01.

## Slide 6
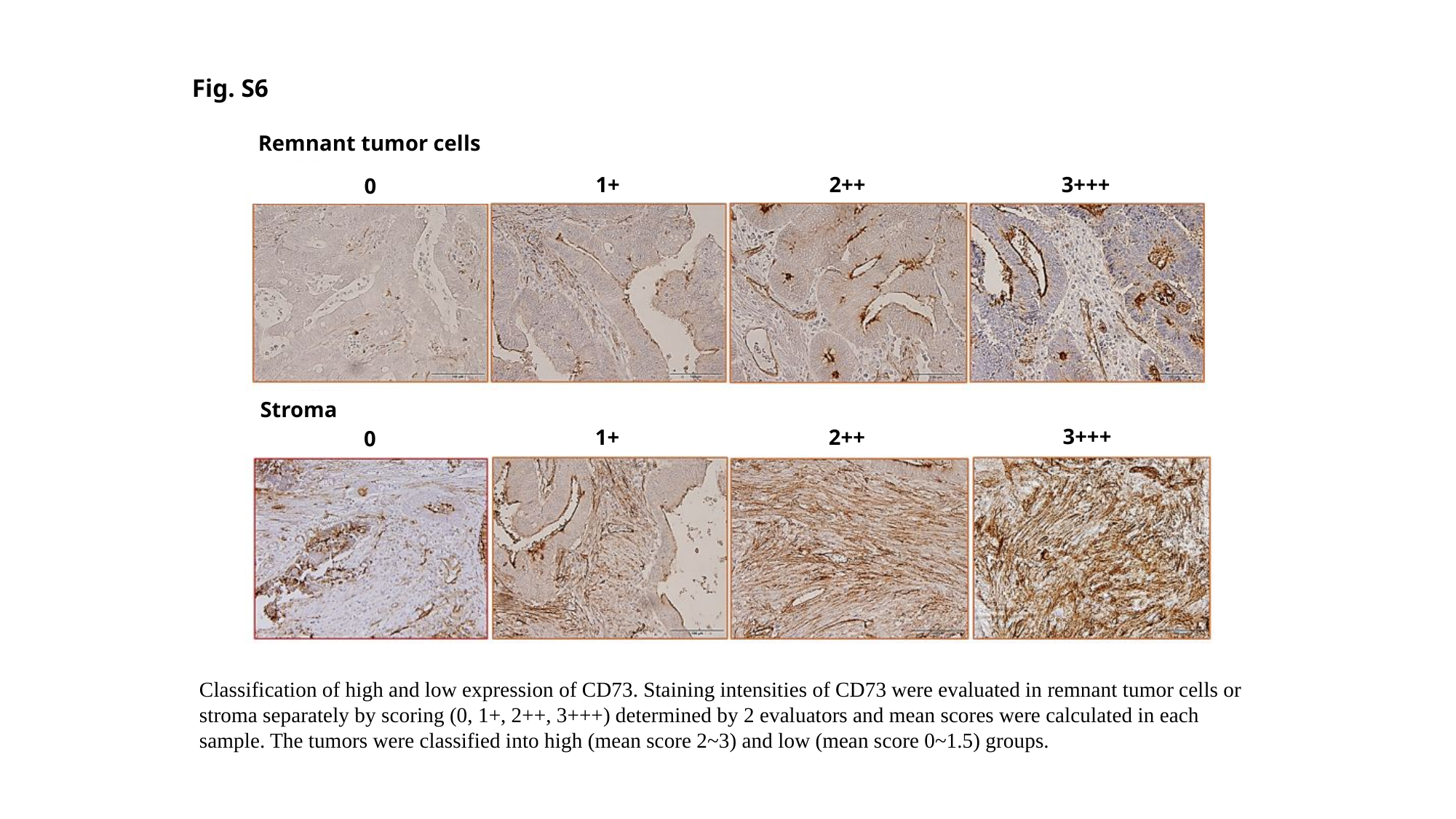

Fig. S6
Remnant tumor cells
3+++
1+
2++
0
Stroma
3+++
1+
2++
0
Classification of high and low expression of CD73. Staining intensities of CD73 were evaluated in remnant tumor cells or stroma separately by scoring (0, 1+, 2++, 3+++) determined by 2 evaluators and mean scores were calculated in each sample. The tumors were classified into high (mean score 2~3) and low (mean score 0~1.5) groups.

## Slide 7
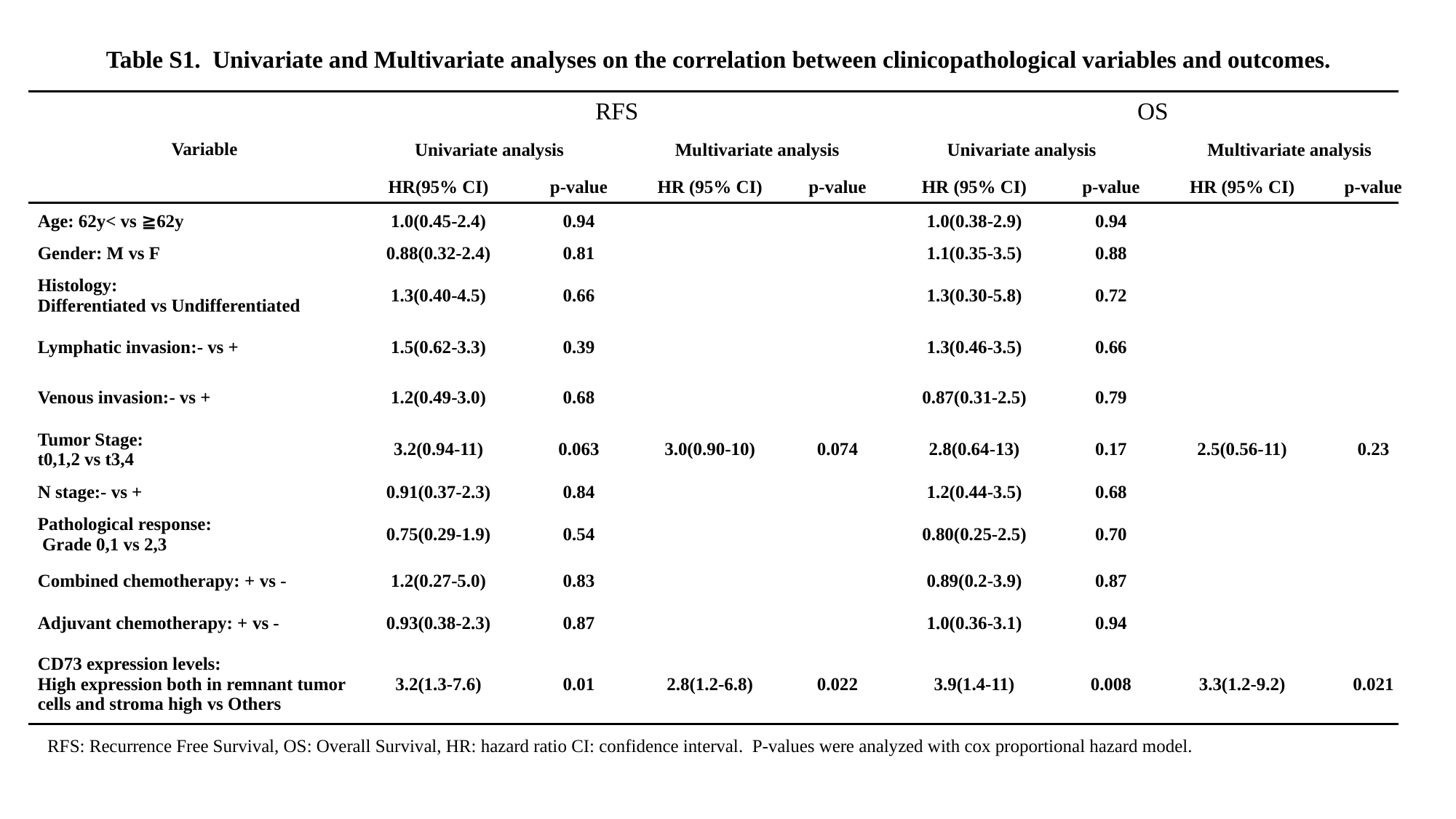

Table S1. Univariate and Multivariate analyses on the correlation between clinicopathological variables and outcomes.
| Variable | RFS | | | | OS | | | |
| --- | --- | --- | --- | --- | --- | --- | --- | --- |
| | Univariate analysis | | Multivariate analysis | | Univariate analysis | | Multivariate analysis | |
| | HR(95% CI) | p-value | HR (95% CI) | p-value | HR (95% CI) | p-value | HR (95% CI) | p-value |
| Age: 62y< vs ≧62y | 1.0(0.45-2.4) | 0.94 | | | 1.0(0.38-2.9) | 0.94 | | |
| Gender: M vs F | 0.88(0.32-2.4) | 0.81 | | | 1.1(0.35-3.5) | 0.88 | | |
| Histology: Differentiated vs Undifferentiated | 1.3(0.40-4.5) | 0.66 | | | 1.3(0.30-5.8) | 0.72 | | |
| Lymphatic invasion:- vs + | 1.5(0.62-3.3) | 0.39 | | | 1.3(0.46-3.5) | 0.66 | | |
| Venous invasion:- vs + | 1.2(0.49-3.0) | 0.68 | | | 0.87(0.31-2.5) | 0.79 | | |
| Tumor Stage: t0,1,2 vs t3,4 | 3.2(0.94-11) | 0.063 | 3.0(0.90-10) | 0.074 | 2.8(0.64-13) | 0.17 | 2.5(0.56-11) | 0.23 |
| N stage:- vs + | 0.91(0.37-2.3) | 0.84 | | | 1.2(0.44-3.5) | 0.68 | | |
| Pathological response: Grade 0,1 vs 2,3 | 0.75(0.29-1.9) | 0.54 | | | 0.80(0.25-2.5) | 0.70 | | |
| Combined chemotherapy: + vs - | 1.2(0.27-5.0) | 0.83 | | | 0.89(0.2-3.9) | 0.87 | | |
| Adjuvant chemotherapy: + vs - | 0.93(0.38-2.3) | 0.87 | | | 1.0(0.36-3.1) | 0.94 | | |
| CD73 expression levels: High expression both in remnant tumor cells and stroma high vs Others | 3.2(1.3-7.6) | 0.01 | 2.8(1.2-6.8) | 0.022 | 3.9(1.4-11) | 0.008 | 3.3(1.2-9.2) | 0.021 |
RFS: Recurrence Free Survival, OS: Overall Survival, HR: hazard ratio CI: confidence interval. P-values were analyzed with cox proportional hazard model.
